# Ethics and Meditation: A New Educational Combination to Boost Verbal Creativity and Sense of Responsibility

**DOI:** 10.3390/jintelligence11080155

**Published:** 2023-08-07

**Authors:** Hélène Hagège, Mohammed El Ourmi, Rebecca Shankland, France Arboix-Calas, Christophe Leys, Todd Lubart

**Affiliations:** 1 FrED, Université de Limoges, UR 20199, 87000 Limoges, France; mohammed.el-ourmi@etu.unilim.fr; 2 Laboratoire DIPHE, Université Lumière Lyon 2, CEDEX 07, 69365 Lyon, France; 3 Institut Universitaire de France, CEDEX 05, 75231 Paris, France; 4 LIRDEF, Université de Montpellier, 34000 Montpellier, France; 5 CRPSI, Université libre de Bruxelles, 1050 Bruxelles, Belgium; christophe.leys@ulb.be; 6 LaPEA, Université Paris Cité & Univ Gustave Eiffel, 92774 Boulogne Billancourt, France; todd.lubart@parisdescartes.fr

**Keywords:** responsibility, education, meditation, mindfulness, creativity, ethics, soft skills, quasi-experimental study

## Abstract

Both creativity and responsibility are important higher-order skills to develop to meet the challenges of the Anthropocene, and both are related to attentional states of consciousness and to ethics. Meditation is a set of practices that trains attentional and emotional regulation. A few studies have shown that different kinds of meditation can foster different kinds of creative thinking, and others have begun to investigate the effect of the combination of meditation and ethics on ethical characteristics (but not yet on creativity or precisely on responsibility, so far). Here, we present a nonrandomized trial with an active control group among second-year science university students (n = 84) to test the effect of the secular Meditation-Based Ethics of Responsibility (MBER) program on creative potential, self-reported awareness, and sense of one’s own responsibility. The results show a large effect of the program on sense of one’s own responsibility and convergent and divergent creative writing tasks, both in conceptual–semantic and engineering-like verbal ideation. They also suggest that convergent conceptual–semantic thinking might moderate the effect of the MBER program on the awareness and sense of one’s own responsibility. This work opens up new research and educational perspectives linked to necessary behavioral changes in the Anthropocene.

## 1. Introduction

In the time of the Anthropocene, warning signals indicating the urgency of changing our modes of functioning are increasing. The latest report from the Intergovernmental Panel on Climate Change (IPCC) highlights the growing vulnerability of human beings, natural species, and regions of the world to global warming, undoubtedly caused by human activity (IPCC 2022). Limiting its effects is still in our power and implies behavioral change (https://www.ipbc.science/, accessed on 12 July 2023). Thus, democratic solutions are an ethical matter that imply (i) individuals feeling committed and being involved in acting ethically and (ii) new and efficient solutions. How can education foster such change to more ethical modes of functioning? This is the general issue that we address here.

Creativity (Division of Mental Health 1993) and ethical competencies, such as responsibility (Sauvé 2000) or wisdom (Sternberg 2018), have been identified worldwide as important skills to develop in order to face the challenges of our century. So, schools and universities should do more to favor the development of these skills to complement the development of higher-order cognitive abilities such as intelligence on which they tend to focus (Sternberg 2018). We have contributed to drafting a national French guide to design trainings about “Sustainable Development & Corporate Social Responsibility Skills” at the university level (CPU and CGÉ 2018). This guide considers creativity, particularly in the service of the “responsibility and ethical skills” (among four other skills). These higher order skills rely on attentional skills (Rebecchi and Hagège 2022; Hagège 2019), and meditation has been shown to constitute a set of attentional training practices in order to favor human flourishing (Dahl et al. 2020). However, the links between creativity and responsibility or ethics and the ways to enhance them through meditation have little been studied so far. These considerations motivated the current study.

### 1.1. What Are the Relationships between Creativity and Responsibility?

We present here an ongoing reflection in order to better situate our study in relation to the issue of cognitive higher-order abilities in the Anthropocene. N.B.: We consider abilities as one of the three dimensions of skill, along with knowledge and dispositions (Mikolajczak and Bausseron 2013).

#### 1.1.1. Definitions of Creativity and Responsibility in Regard to Higher-Order Cognitive Skills

Responsibility and creativity have been conceptualized in many ways.

Creativity is nowadays defined, generally, as the ability to produce things or ideas that are new and adapted to their context (Runco and Jaeger 2012). This process is characterized by two complementary ways of thinking. First, divergent thinking is an exploratory step where different solutions of a contextualized problem are imagined. Second, convergent thinking allows the integration of different relevant elements into a single adapted and innovative response (Lubart 2001). Creativity is considered as largely domain-specific: it can be a cognitive higher-order ability in math or a procedural (i.e., motor) one in painting, for instance. Also, different levels of creativity have been distinguished: mini-c (thoughts and mental patterns typically involved in learning), Little-c (in daily life actions such as cooking at home), Pro-C (professional creativity such as architects at work), and BIG-C (eminent cases) (Beghetto and Kaufman 2014). We postulate that the creativity most needed to solve the problems inherent in the Anthropocene concerns more the inventiveness related to everyday life, the ability to change functional and behavioral habits (notably towards greater energy and material sobriety), to promote sustainable degrowth, than revolutionary technological ideas—which could nonetheless help turn the tide towards sobriety.

Responsibility has been theorized in distinct fields of research such as moral psychosociology (Swaner 2005), moral philosophy (Knobe and Doris 2010), phenomenology, and ethics (Simon 1993). This notion is consubstantial with the ones of education and ethics, as it is a central aim of both processes (Paturet 2003). It literally means “the ability to respond” (response-ability). Initially a legal concept of imputability referring to the fact of being accountable for oneself, it extends, in ethics, to the capacity to be answerable for who or what is vulnerable (Prairat 2012): a majority of authors insisted on a particular way of relating to others (Gendron and Bouchard 2015), but Jonas, one of the greatest ethicists of the last century, insisted rather on humans’ relationships to scientific–technical developments that can harm nature (Jonas 1990). We integrated the two foci, proposing that responsibility involves a harmonious response to the situation, which optimally takes into account self, others, and the nonhuman environment (Hagège 2019). This ability relies on the awareness of the consequences of one’s actions on the world and an interest in taking them into account (sense of one’s own responsibility—SOOR). It also involves the awareness of one’s limits and “shortcomings” and their consequences on one’s environment (awareness of one’s own responsibility—AOOR; Hagège 2022).

Thus, in addition to the answerability, responsibility implies a commitment to making decisions and acting appropriately on the basis of a thorough analysis and understanding of the situation and its needs (Lacroix et al. 2017). So, it would rely on higher-order cognitive abilities such as critical and complex thinking (Hagège forthcoming). But the links with creativity are less theorized.

#### 1.1.2. Links between Creativity, Responsibility, and Ethical Skills

Strikingly, in the original formulation of life skills (Division of Mental Health 1993), creative thinking is considered as an internal resource to *respond* adequately to a situation:

“Creative thinking contributes to both decision making and problem solving by enabling us to explore the available alternatives and various consequences of our actions or non-actions. It helps us […] to respond adaptively and with flexibility to the situations of our daily lives”.

(Division of Mental Health 1993, p. 2)

However, in creativity research, the links between creativity and responsibility have empirically been mainly indirectly studied to date through the investigation of outcomes close to responsibility. First, the fact that creativity can serve evil aims has been stressed: it has been shown that creative people can sometimes have low integrity or use their skill to voluntarily harm people (Cropley et al. 2013). Moreover, despite good intentions motivating numerous creative acts, unforeseen deleterious consequences can emerge in the complex systems in which we are embedded (Cropley et al. 2010). So, creativity is not a guarantee of morality. However, creativity and ethics cross paths in multiple manners (Moran et al. 2014). For instance, the mode of creative imagination seems all the more ethical as the individual has benefited from a secure attachment as a child, and the more or less ethical modes of functioning can be neurologically correlated (Narvaez and Mrkva 2014).

So, researchers have increasingly questioned the ethical and intentional outputs of creative productions in the last decades and unethical outcomes (“dark sides”) of creativity have been noted (Cropley et al. 2010), whereas “transformational creativity” (Sternberg 2021b) “with full integrity” (Sternberg and Lubart 2023) or “responsible creativity” (Rebecchi and Hagège 2022) have been proposed as ways to characterize the orientation of creativity towards ethical actions, i.e., towards the “common good” (Sternberg 2021a).

**Figure 1:**
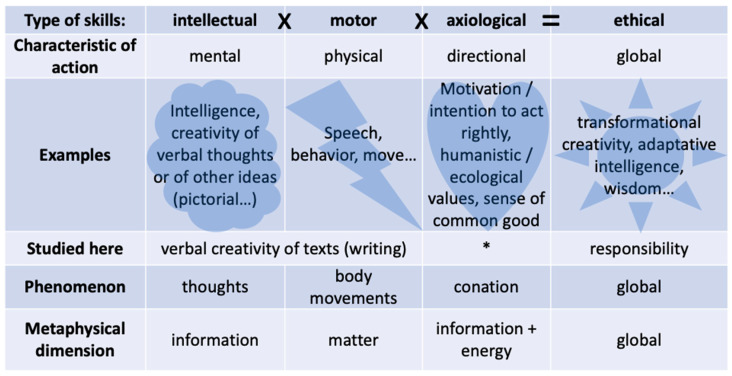
Part of the equation involving creativity and responsibility in regard to ethical skills. *Note*. The equation at the top is a multiplication: if one of the factors is zero, then the result is zero. This equation is incomplete because responsibility and ethical competence—in which responsibility is central—also require other “heart”-type skills (namely emotional, relational, and attentional skills; Bernatchez 2022; Hagège 2019; Lacroix et al. 2017). So, we consider ethical skills as higher-order skills of a complex or multidimensional type. Motor skills are usually called “procedural” skills in the literature, but as this word refers to a sequence of predetermined actions, we rather use the adjective “motor” here, which is more inclusive of creative actions. Also, in our theoretical framework, intellectual skills are included in a wider category of “epistemic” skills that also include lower-order cognitive skills, such as cognitive flexibility (Hagège 2019, forthcoming). * In the present study, responsibility is assessed by a self-reported instrument that as such indicates an axiological—which means “related to values”—skill more than a properly ethical one (Appendix A). For the notion of metaphysical dimension, see Hagège (2019).

Also, in different types of ethics, for example virtue ethics (MacIntyre 2001) and organizational ethics (Lacroix et al. 2017), intellectual skills are considered as internal resources in the service of ethical skills (or “moral virtues”), such as responsibility. So, higher-order abilities such as intelligence and creativity might be required to act rightly in the Anthropocene, but they are not sufficient, they also must be (i) oriented towards a common good and (ii) translated into physical actions (Figure 1; Sternberg 2018). So, ethical skills such as wisdom (Sternberg 2018), “successful” (Sternberg 2018), or “adaptative” intelligence (Sternberg 2019) and responsibility (Hagège 2019) may achieve this coordination of intellectual skills with ethical physical actions: they involve multiple types of skills; so, they are more complex than creativity, which can be solely intellectual or motor, depending on the domain (Figure 1), and they necessarily involve the “practical intelligence” or “common sense” that applies such higher-order cognitive abilities to “real-world contexts” (Sternberg 2018, p. 4; Lacroix et al. 2017).

Notice that in the field of ethics, the term “creativity” is quite rarely mentioned. It is explicitly mobilized in the ethics of authenticity, where it refers to an innovative way of living the contingency of one’s own choices and actions (Flynn 2001). However, in organizational ethics, it is only implicitly referred to as a characteristic of the response, which breaks away from “repetition” or “automatic conformity”, and thus implies, in connection with reflexivity, “an ability to extract oneself from one’s own routines” and “stereotyped and thoughtless actions” (Lacroix et al. 2017, pp. 107–8, personal translation). In this line, to us, responsibility is a matter of creating an ethical “production” in the widest sense—be it oral as a speech or material for instance—to the detriment of past cultural conditioning and personal psychological predetermined schemes, such as thought–action repertoires (Chambers et al. 2009), which, by definition, cannot integrate the novelty of the present complexity. So, we assume that creativity and responsibility are different types of higher-order skills needed in the Anthropocene: linked—responsibility requires creativity—but not correlated (Figure 1).

Now, as far as educational needs in the Anthropocene are concerned, skills such as responsibility and creativity are more lacking than mental intelligence, which is already a major focus of our Western schools (Sternberg 2018). So, how could education improve creativity and responsibility?

### 1.2. Can Meditation Enhance Creativity and Responsibility, and How?

Both the higher-order skills of creativity (reviewed by Rebecchi and Hagège 2022) and responsibility (Hagège 2019) rely on attentional states of consciousness, such as mindfulness, which can be typically enhanced by meditation (see below). And some authors hypothesized that meditation or mindfulness training could enhance responsibility (Hagège 2019, forthcoming), ethical sensitivity (Bernatchez 2022), or ethical actions (discussed by Monteiro et al. 2015, and by Purser 2015).

#### 1.2.1. Origins and Definitions of Meditation and Mindfulness

Meditation is a set of heterogeneous practices that trains attentional and emotional regulation (Lutz et al. 2008) and favors well-being and human flourishing (Dahl et al. 2020). They originate from spiritual disciplines, notably Buddhism and yoga, which means “unity” or “oneness” in Sanskrit. Traditional *aṣṭāṅga-yoga* is composed of height branches: only one concerns the popular dynamic physical postures (*āsanas*), whereas the two first establish ethical principles, and the last half deals with meditation or meditation-triggered states of consciousness (Patañjali 1991), with some developmental stages of these states having been documented in the scientific literature (Lutz et al. 2007; Grabovac 2015). Also, different types of meditation have been distinguished: focused attention (FA), open monitoring (OM) and loving-kindness (LK) meditations (Lutz et al. 2008). FA meditation aims at training endogenous attention to focus on a support such as one’s breath (e.g., to try staying constantly aware of breathing, identifying any distractions such as sleepiness or mind wandering and bringing attention back to the breath as soon as possible). OM meditation tends to develop a wider attentional focus, in which the person tries to be simultaneously aware of all the perceptible phenomena, whether they are sensory or mental. Finally, LK meditation cultivates qualities such as universal love and compassion. More recently, an enriched categorization of meditation has been proposed: awareness-based (among which FA and OM meditations are situated), connection-based (including LK meditations), purpose-based (designed to foster clarity and embodiment of intrinsic values and aims), and insight-based (eliciting self-knowledge and self-inquiry) (Dahl et al. 2020).

Now, stemming from *Theravāda* Buddhism, mindfulness meditations were secularized. Mindfulness was initially defined as “the awareness that emerges through paying attention on purpose, in the present moment, and nonjudgmentally to the unfolding of experience moment by moment” (Kabat-Zinn 2003, p. 145). Its definition has been enriched at the margin later (ex: Bishop et al. 2004). It has been popularized thanks to the Mindfulness-Based Stress Reduction (MBSR) program, which was initially dispensed in the clinic to promote remission in recurrent depressive subjects or with chronic pain and is nowadays probably the mostly taught and studied secular meditation program worldwide. This program contains OM and FA meditations.

#### 1.2.2. Effects of Meditation on Creativity, Functional Change, and Ethics-Related Variables

It seems that different kinds of meditation can foster different kinds of creativity. Indeed, FA meditation tends to promote convergent thinking, whereas OM meditation (such as Integrated Body Mind Training, Ding et al. 2014) would rather favor divergent thinking (reviewed by Lippelt et al. 2014). Also, (awareness/) mindfulness-based programs, which include both FA and OM training, could improve verbal creativity, as it has been shown with randomly and waiting-list-controlled trials (Bellosta-Batalla et al. 2021). Moreover, divergent thinking is greater among mid- to long-term mindfulness practitioners than among novice ones, and this expertise is reflected in neural correlates that indicate weaker mind wandering (Berkovich-Ohana et al. 2017)—an ordinary attentional state of consciousness that is expected to decrease with meditation training (Lutz et al. 2008). Finally, there are several attentional similarities between creativity and the mindfulness attentional state (reviewed by Rebecchi and Hagège 2022).

Now, as far as the link between meditation and ethics is concerned, data are more complex.

First, the effect of meditation on higher-order cognitive skills has been very little investigated to date. Some evidence suggests that mindfulness training might improve critical thinking (Ritter-Williams et al. 2022), but mainly for open-minded individuals in need of cognition (Noone et al. 2016). Also, meditation could help in becoming better at math in high school (reviewed by Waters et al. 2015) and at a comprehension reading task in university (Mrazek et al. 2013). But these effects have to be confirmed. Also, because motor skills are central to ethics (Figure 1), we problematize education for responsibility in the Anthropocene in terms of change in mode of functioning, including behavior (see above). Importantly, meditation programs have proven to be efficient for such changes in regard to different health issues. For instance, mindfulness meditation has been shown to be efficient to foster behavioral regulation (reviewed by Keng et al. 2011) and to treat behavioral disorders, such as sexual (reviewed by Mize 2015), gambling (meta-analyzed by Maynard et al. 2015), or eating (reviewed by Mantzios and Wilson 2015; Olson and Emery 2015) disorders. As far as the involved mechanisms are concerned, emotional skills such as emotion regulation seem to constitute a major lever of behavioral change (Hölzel et al. 2011; Chambers et al. 2009; Cottraux 2007). Moreover, the most robust statistically measured effect of mindfulness programs concerns precisely such skills and relationships with others (meta-analyzed by Sedlmeier et al. 2012). That is why, nowadays, cognitive behavioral therapies concentrate on this skill and tend to use meditation to develop it (Hölzel et al. 2011; Chambers et al. 2009; Cottraux 2007). Evidence shows that as predicted, meditation enhances attentional skills (meta-analyzed by Sumantry and Stewart 2021; Lutz et al. 2008; reviewed by Braboszcz et al. 2010), which are assumed to interact with emotional skills: once emotions become an object of mindful attention, they can be effectively regulated thanks to (i) a neurologically based inhibition of automatic reactions (Lutz et al. 2008)—this could explain how meditation could favor creative functioning—and (ii) several psychological processes, such as metacognitive insight, a decrease in experiential avoidance (reviewed by Chambers et al. 2009), and reappraisal or extinction of stressful emotions (Hölzel et al. 2011). So, meditation seems to have the potential to improve two types of skills (attentional and emotional, only evoked in the note of Figure 1) that could contribute to motor and thus ethical skills. Along this line, in ethics of care and in phenomenology, some authors consider attention as a major skill that corresponds to the ability to perceive the whole (human and nonhuman) environment lucidly and lovingly (Bernatchez 2022). Also, emotional skills are recognized as positive levers of ethical skills that enable positive relationships to others (Sternberg 2021a; Bernatchez 2022).

Second, in the same vein, one meta-analysis showed that LK meditations can foster prosocial behaviors (Luberto et al. 2018). Two meta-analyses supported the proposal that mindfulness increases prosocial behavior (Berry et al. 2020; Donald et al. 2019; discussed by Schindler and Friese 2022). Particularly, mindfulness-based programs without explicit ethical teaching increased compassionate helping and reduced behaviors related to prejudice or retaliation, but not instrumental or generous helping (Berry et al. 2020), which we do not all equate with “ethical actions”, but which often are presented as such in the literature. However, some studies suggest that such a prosocial effect might be restricted to people with certain predispositions, for instance, with interdependent self-construal (either preexisting or primed), which corresponds to operational collectivist values (Poulin et al. 2021) or high levels of dispositional empathy (Chen and Jordan 2020). Moreover, mindfulness practice seems, on the contrary, to decrease prosocial behavior among people with independent self-construal (either preexisting or primed), which corresponds to operational individualistic values (Poulin et al. 2021), as well as among less empathetic individuals (Chen and Jordan 2020). However, as shown with an online short meditation training, this unexpected effect is not observed if the training includes explicit ethical instructions (Chen and Jordan 2020). So, there is a need to clarify the effect of (mindfulness) meditation on such variables, given the initial skills or dispositions of the subjects and the inclusion or the exclusion of explicit ethical content in the meditation-based program. Also, several studies showed that trait mindfulness is linked to proenvironmental behavior (Geiger et al. 2018; Panno et al. 2017; Wamsler and Brink 2018) and connectedness to nature (Howell et al. 2011; Wolsko and Lindberg 2013). Furthermore, meditation practitioners expressed a greater inclination towards sustainability than nonpractitioners (Jacob et al. 2008; Loy and Reese 2019).

Third, as far as an explicit link between ethics and meditation is concerned, there are some debates in the literature (ex: Purser 2015; Monteiro et al. 2015), and we address here only empirical data. Even if the literature is still scarce, some evidence suggests that explicitly ethically oriented meditation might be more effective to enhance prosocial behavior than health-oriented meditation (Chen and Jordan 2020), which could nevertheless improve ethical decision making (Shapiro et al. 2012).

So, altogether, meditation seems to have the potential to enhance responsibility, and we report elsewhere empirical arguments in favor of a positive effect of meditation on responsibility (Hagège forthcoming)—an effect which has not been studied yet per se to our knowledge.

### 1.3. Scope of the Present Study

The present article will shed, as far as we know, new light on several points. First, the relationships between creativity and responsibility remain unclear, and we analyze the statistical significance of this link here for the first time. Second, we present the first validation study of a concrete educational initiative that would target the joint development of creativity and responsibility, which seems to be relevant to fostering a favorable evolution of our world, as we argued above. Third, authors have stressed the importance to explicitly include an ethical dimension in meditation-based programs in order to foster ethical development (Lomas 2017; Thupten 2019; Condon 2019)— to thus meet the challenges of the Anthropocene by enhancing axiological skills as well (Figure 1)—and we did not find any study of the impact of a structured ethically oriented meditation-based program on creativity. We developed such a program, named the Meditation-Based Ethics of Responsibility (MBER) program, and investigated both latter points. Fourth, as far as the effect of meditation on (self-reported) responsibility per se is concerned, the present article is seemingly the first contribution. Finally, the present contribution offers a quasi-experimental study about the MBER program.

Indeed, we present here the procedure and results of a nonrandomized and actively controlled trial that aimed at assessing the impact of the MBER program on awareness and sense of one’s own responsibility and verbal divergent and convergent creative potentials. Our research questions “Qn” and corresponding hypotheses “Hn” are the following:

(Q1) Does the MBER program increase creativity scores (here, the conceptual–semantic and engineering-related and convergent and divergent verbal creative potentials)? H1: Yes, it does.

(Q2) Does the program increase responsibility scores (i.e., the awareness and sense of one’s own responsibility scores)? H2: Yes, it does.

(Q3) Does the program decrease responsibility scores on a subsection of the population identified according to their initial creativity score and thus have “unwanted effects”? H3: No, it does not.

(Q4a) Is there a correlation between creativity and responsibility scores at the pretest measurement? (Q4b) Do the creativity scores mediate or moderate the effect of the program on responsibility? We expected that creativity scores on the pretest would mediate or moderate the effect of the program on responsibility scores at the post-test (H4b). Also, we hypothesized that creativity scores were independent of responsibility scores (so no correlation was expected between the two; H4a), because the latter should also rely on other necessary dimensions (Figure 1).

## 2. Materials and Methods

### 2.1. The MBER Program

We designed a 25 h mindfulness-based program called MBER derived from an existing program, which was itself based on early forms of MBSR (Mindfulness-Based Stress Reduction; Kabat-Zinn 1990) and MBCT (Mindfulness-Based Cognitive Therapy; Segal et al. 2002). These initial programs are dedicated to attentional and emotional training. The MBER program is detailed in a textbook (Hagège 2022). Briefly, it promotes open-mindedness as well as an ethical orientation through a reflection on values. Furthermore, in line with an ethics of responsibility (Hottois 1996), it proposes practices to become more aware of the consequences of one’s own actions, thoughts, and emotions on self, others, and the nonhuman environment. It incorporates the four above-mentioned categories of meditation (awareness-, connection-, purpose-, and insight-based) and articulates them with secular philosophical and psychological knowledge on one side and exercises—which are not meditations per se—that foster reflexivity and insight on the other side. Participants are also encouraged to practice for 20 to 40 min on their own between sessions. The themes of the six 3 h sessions and the whole silent day, respectively, are the following: (1) ethics, values, attention, and automatic pilot; (2) stress reduction and feeling guilt; (3) dual modes of functioning, mental judgements, and nonduality (kind of wisdom); (4) content and mechanisms of thoughts (links with emotions), language and cultural biases; (5) empathy, relatedness with the nonhuman environment, emotion regulation and integration; (6) joy, authenticity, and creativity; (7) (silent day) care, gratitude, interdependence, and benevolence.

### 2.2. Participants

We conducted a quasi-experimental study (pretest/post-test with control group) with young adults (n = 105) in the context of a French faculty of “hard” sciences. The MBER program was part of the students’ curricula as a module of “general knowledge”, which they could choose among other modules. The experimental group was composed of university science students who chose the MBER module, whereas the control group consisted of students who chose other 25 h modules of “general knowledge” related to the hard sciences but not to ethics, creativity, or meditation. Because the students chose their optional module through an online procedure, we had the means to contact them only after this choice. It was thus not possible to assign them randomly to a group. Inclusion criteria were to agree freely to take part anonymously in the study and, for the analyses of the experiment, to complete both pretest and post-test measures. Anonymity was guaranteed, and data between pretest and post-test were matched using an anonymous code created by each participant in an imposed manner, based on data to which we did not have access to (first 2 letters of their fathers’ and mothers’ first names, etc.). Twenty participants were absent at the post-test. They did not significantly differ from the remaining participants in age and sex. Also, one participant was removed from the analysis because her responses appeared to be an extreme outlier for the majority of variables (thus, we suspected a lack of sincerity). Finally, we retained 48 (M_age_ = 20.48, SD = 1.60) and 36 (M_age_ = 20.18, SD = 1.16) participants in the experimental and control groups, respectively (n = 84). We kept participants who did not completely fill in all relevant measures.

### 2.3. Measures

We used the following set of new tools.

A 6-point “Awareness and Sense of One’s Own Responsibility” Likert scale (Appendix A) was developed for the purpose of this study and is currently being validated (Hagège et al., in prep.). It is thus a preliminary self-reported instrument that is designed to assess variables that indicate responsibility. It contains two subscales that emerged from an exploratory factor analysis, with varimax rotation explaining 25.55% of variance with a first factor and 20.65% of the variance with the second: respectively, the sense of one’s own responsibility (SOOR) with 7 items, such as “I feel concerned by the fact that my actions contribute to a better world” and awareness of one’s own responsibility (AOOR) composed of 3 items, such as “I am aware of the consequences of my limitations and defaults on my environment and on others” (Appendix A). The internal reliabilities were satisfactory, both at the pretest (n = 105, Cronbach’s α = 0.73 for SOOR, 0.81 for AOOR, and 0.70 total) and the post-test (n = 84, Cronbach’s α = 0.77 for SOOR, 0.79 for AOOR, and 0.77 total). We refer to the scale by the term “Responsibility” in the tables of the article. On the questionnaire, it was indicated that the topic of the scale concerns “your relationship with the world”.

We used tasks based on the EPoC (Lubart et al. 2011) that measure the creative potential in two domains: verbal conceptual–semantic and engineering-like, with both convergent-integrative and divergent-exploratory forms. We adapted tests that exist for children to adults. As far as the divergent conceptual–semantic test is concerned, participants had 5 min to write as many original sentences as they could that contained an imposed word (example: “gas”). For the convergent conceptual–semantic test, they had 5 min to indicate an original word that linked 3 unrelated imposed words (example: “anemone, itinerary, superiority”) and explain this link through one sentence. As far as the engineering-like tests are concerned, the participants had to imagine a game for children by combining 3 imposed objects (named and also shown as pictures; example: a magnifying glass, a ruler, and masking tape) for the convergent thinking part. And they were expected to write as many possible original uses of an ordinary object (named and also shown as picture; example: a pen) for the divergent thinking part (alternate uses task). The conceptual–semantic and engineering-like tests are designated, respectively, by “C-S” and “E-L” in the tables and are preceded by “C” or “D” if convergent or divergent. We used two different forms of each test (A and B): each individual completed a different version at the pretest and the post-test, and A and B forms were randomly assigned at the pretest in both experimental and control groups. The four raters read carefully through the EpoC test guide and harmonized their understanding of it through discussion before scoring the productions. They independently noted the creativity of each production on a 7-point Likert scale, blind to the production’s group of origin (more details are available in the EPoC test guide; Lubart et al. 2011).

Each individual score is the mean of these four notations. The interjudge reliabilities of the 4 creativity subscores at pretest and post-test range, according to Fleiss’s Kappa, from 0.22 (fair) to 0.45 (moderate), with an average of 0.37 (sd = 0.07). Given the satisfactory bivariate correlations between the 4 scores of creativity, both at pretest (0.23 < r < 0.53; *p* < .05; see below for more details) and post-test (0.35 < r < 0.57; *p* < .003), we calculated a global verbal creativity score (labeled “creativity” in the tables) which had satisfying internal reliabilities, both at the pretest (Cronbach’s α = 0.71) and the post-test (Cronbach’s α = 0.77).

### 2.4. Administration Procedure and Statistical Analysis Software

We administered these paper and pencil instruments in the context of a larger-scale research project. The assessment of creativity lasted 20 min (4 × 5 min) and occurred before the administration of the questionnaire, which included other self-reported scales that are not presented here (same procedure and content for all subjects of the experiment). The title of the questionnaire sheet was “questionnaire to be completed as part of a scientific study of modules of general knowledge”, and then it was written that “this questionnaire is divided into sections on different themes” (the themes were vague and indicated as subtitles). Then, we conducted the statistical analyses using SPSS Statistics 20.0.0 software. Moderation and meditation analyses were conducted using the Hayes’ module of SPSS.

## 3. Results

### 3.1. Analysis of the Scores and of Their Comparative Evolutions

#### 3.1.1. Descriptive Statistics of the Sample

We first assessed the means of the variables of interest in the experimental and control groups (Table 1).

Initial creativity scores were below the central point of the scale (<4), whereas responsibility pretest scores were above it (>3.5). Therefore, the first ones had more room for improvement than the second, on average.

#### 3.1.2. Assessment of the Impact of the MBER Program on Indicators of Creativity and Responsibility

As both groups did not a priori present the same motivation to learn meditation and, moreover, had different sex ratios, we controlled the pretest scores thanks to a one-way between-groups analysis of covariance (Table 2). So, the scores at the pretest were used as the covariates of the post-test scores. Preliminary analyses confirmed the absence of violation of the assumptions of normality, linearity, homogeneity of variances, and homogeneity of regression slopes.

The program showed large effect sizes on both variables: Awareness and Sense of One’s Own Responsibility (through SOOR) and verbal creative potential (convergent, divergent, engineering-like, or conceptual–semantic), significant at a level below 0.003. AOOR is the only variable for which we observed no significant change below the threshold of 5%. All the changes that are significant show medium to large effect sizes and are in the expected direction (rising on average between the pretest and the post-test). These results show a strong effect of the MBER program on indicators of creativity and responsibility, suggesting its efficiency on both skills (H1 and H2).

### 3.2. Relationships between the Variables Indicating Creativity and Responsibility

As the relationships between creativity and responsibility have not been studied earlier, we explored them, keeping in mind that our measure of responsibility is preliminary.

First, the correlations between all scores and subscores for all participants of the pretest indicated that only creative potential scores are significantly intercorrelated (Table 3). This tends to suggest that creative potential has seemingly nothing to do with initial responsibility, as we expected (H4a).

We then assessed if it would be plausible for creativity scores to have the unwanted effect of negatively affecting the evolution of the responsibility scores between the pretest and the post-test in a sub-section of the experimental group (Table 4).

We did not observe such an unexpected effect in either of the groups characterized by their initial amount of creativity (Table 4). The means of Awareness and Sense of One’s Own Responsibility are similar between both halves—t(44) = 0.33, *p* = .74—and the most important rises (large effects) concern the creativity scores among the participants who initially had the lowest scores. This effect is specific to the experimental group, because no significant increase in these scores is observed in any median split half of the control group. It is striking to note that the only scores that do not increase significantly at the 5% threshold are the scores of responsibility among the participants who have the initially lowest creativity scores (and also the subscores AOOR and SOOR in the other half). This suggests that creativity might moderate the effect of the MBER program on responsibility.

For this reason, we tested this moderation effect but did not find any significant moderation model at the 5% threshold. However, by testing the moderation effects of the different creativity subscores independently, one model was found to be consistent (*p* less than .06) (Table 5).

Table 5 shows that when the convergent conceptual–semantic creativity score is low (1.77) at the pretest, the responsibility score does not significantly increase for the subjects who followed the MBER program (*p* = .091). However, when this initial score is higher (3.17 or 4.57), the rise in the responsibility score becomes significant (*p* < .001). This result suggests that the initial score of convergent conceptual–semantic creativity might moderate the effect of the MBER program on Awareness and Sense of One’s Own Responsibility, but this should be confirmed in another study.

We also tested if pretest creativity scores might mediate the effect of MBER program on responsibility post-test scores, and it did not yield any significant result.

## 4. Discussion

We conducted a nonrandomized actively controlled experiment in order to assess the impact of the MBER program on indicators of creativity and responsibility and to explore the potential relationships between both variables.

### 4.1. Effect of the MBER Program on Indicators of Creativity and Responsibility

In the context of our study, our first hypothesis (H1) is largely supported, because we observe large size significant effects of the MBER program on convergent and divergent, both conceptual–semantic and engineering-like creative potentials (Table 2). Indeed, the MBER program includes four kinds of meditation (given the nomenclature of Dahl et al. 2020), among which are FA and OM (mindfulness training), which belong to awareness-based training. And, as reviewed in the introduction, both types of meditation have separately or jointly been shown to improve divergent and convergent thinking (reviewed by Lippelt et al. 2014), as well as verbal creativity (Bellosta-Batalla et al. 2021). The amplitude of the effect that we measure is of the same order of magnitude as that obtained by comparing novice mindfulness practitioners with more advanced ones (with a cumulated practice of roughly 900 h to 2000–8000 h, respectively), whereas, in the same study, no significant effect is observed when these novice meditators are compared with a control group (Berkovich-Ohana et al. 2017)—albeit the tools to assess divergent thinking are not the same as those that we used. In our study, subjects meditated for only a few dozen hours (≤50 h), but we only measured short-term effects. First, we interpret that the combination of awareness-based meditations with the three other types of meditation and together with the ethical aim, but also specific knowledge (notably about cultural conditioning, personal psychological predetermined schemes, and ways to emancipate oneself from both processes) and reflection about one’s own functioning, might be more powerful tools to foster creativity (and responsibility) than more uniform approaches. Second, we hypothesize that the particular adaptation of the program (containing theories and scientific references) to the specificity of the participants (science students) might be partly responsible for the magnitude of its effect. Indeed, to our knowledge, mindfulness-based programs have been adapted to specific pathologies or health issues, such as addictions (reviewed by Sancho et al. 2018), depression (Hofmann and Gómez 2017), and so forth, but not to specific healthy dispositions or traits. So, further (medium- and long-term) studies, some involving different populations from the one involved here (students of fine arts or older workers, for example), are needed.

Our H2 hypothesis was only partially confirmed: the effect of the MBER program on the SOOR subscore was of the same type and magnitude as the ones measured for creativity, but AOOR did not significantly change after the program.

The SOOR items concentrate on the ethical intention (axiological skill): to feel concerned by the impact of one’s own actions on the world and to be motivated to orient one’s own actions towards a better impact. To our knowledge, these are the first empirical data of this sort, and the closest study is one which showed that an 8-day online short ethically oriented meditation training enhanced a measure of prosociality (namely the altruistic donation of money to a family in distress), as compared with more classical online mindfulness training (Chen and Jordan 2020). So, there are many other studies to be done in order to further explore our result, its reproducibility, and the conditions of its emergence.

The AOOR items express the awareness of one’s own functioning and actions and of their consequences on oneself and the world: either the program did not increase this awareness or there is a methodology issue here. This first interpretation tends to contradict what the students expressed in informal written feedback that we collected (Hagège 2015, 2022). Also, some published studies support the second interpretation. Indeed, the practice of meditation fosters the awareness of one’s own lack of awareness (Lutz et al. 2008). So, it is possible that the development of this last skill has somehow neutralized an improvement of the AOOR scores (Shankland et al. 2017): participants became at the same time more aware of their functioning and of its consequences, as well as aware that they were far from being fully aware of it. Therefore, this aspect needs further investigation, for example, using in-depth interviews. Also, another potentially more effective way of formulating items from a scale that assesses the impact of the MBER program on the development of responsibility could rather start like this: “*In the past two months, I became more* aware of the consequences of my [functioning]”.

### 4.2. Relationship between Creativity and Responsibility

“Primum non nocere” (first do no harm) is Hippocrates’ first oath. In this vein, we verified the absence of unwanted effects of the program on responsibility depending on initial creativity scores. Participants with the lowest initial creativity made the most progress on this score (Table 4); so, all participants seemed to have benefited from this expected outcome of the program: we did not measure an unexpected effect compared with classical mindfulness meditations without explicit ethical goals (Poulin et al. 2021; Chen and Jordan 2020). So, our hypothesis H3 is confirmed within the limits of the variables that we studied. As confirmed by one study (Chen and Jordan 2020), the ethical orientation of the meditation might be responsible for this characteristic of our results. This interpretation could be challenged by conducting the same study, while replacing all ethical content of the MBER program by another topic (for example self-orientation as in personal development); if we are right, then initial lower scores in responsibility and creativity might decrease, in contrast to those with initial high levels in both sets of variables. We could also test, in the future, the influence of other initial variables (more of the “trait” type), such as the independent or interdependent self-construals studied by Poulin et al. (2021).

As far as our H4 hypotheses are concerned, we supposed that responsibility requires some kind of creativity and that on the contrary, creativity does not require responsibility. Both sets of variables were not significantly correlated with each other (Table 3). So, H4a seems confirmed. Moreover, the two sets of algebraic differences between post-test and pretest scores were not correlated either, which suggests that there was indeed no coevolution of the two sets of variables. Thus, in coherence with the literature that we evoked in the introduction (Sternberg 2018, 2019; Figure 1), creativity and responsibility are at least partly dissociable.

Some preliminary results suggest that convergent conceptual–semantic creativity might moderate the effect of the MBER program on responsibility (Table 5): a minimum initial level of this creative potential might be required for the MBER program to foster responsibility. This would go in the direction postulated in H4b, with responsibility depending on creativity. However, this result should be confirmed.

So, overall, further investigation might be conducted that would overcome some limits of the present study (measure of in situ interactions between creativity and sense of responsibility, solid and more contextual and objective measure of responsibility, randomly controlled trial, etc.).

## 5. Conclusions

This study offers some preliminary explorations of the relationships between creativity and responsibility. It supports the power of the MBER program to foster convergent and divergent verbal ideation coupled with writing (a verbal creativity that we consider as a motor and higher-order cognitive skill; Figure 1), both in conceptual–semantic and engineering-like creative potentials and a sense of one’s own responsibility. Fortunately, the program seems also to be devoid of any counterproductive effects in terms of creativity and responsibility enhancement (because we did not observe any decrease in these variables). Thus, overall, it seems to be a promising educational tool to help change individual functioning in order to address the challenges of the Anthropocene.

In conclusion, it seems that a misconception of human functioning, prevalent in society and even among some scientists (Brandtstädter 2007), suggests that higher-order cognitive skills control functioning (including behavior) according to a simple top-down causality (“I do it because I decided to”; Hagège 2014). However, this model has been widely altered, if not invalidated, in various fields of research (“I’m not aware of everything I do, let alone all the reasons why I do it”; e.g., Bode et al. 2014; Custers and Aarts 2010; Pearson et al. 2009; Haidt 2001), which calls into question the notions of free will (Wegner 2003) and responsibility (“If these processes are unconscious, how can I take responsibility for my functioning?”; King and Carruthers 2012; Faucher 2012; Levy 2014), and thinking about this can make one dizzy. So, as we illustrated in the introduction (see Section 1.1.1 and Section 1.2.2), the relationship between higher-order cognitive skills and more global (ethical) individual functioning is complex; thus, educational solutions need to be more varied than traditional teaching methods (Rebecchi and Hagège 2022). Moreover, we have not added to the discussion the social, economic, and other layers that make the determinants of individual functioning models even more complex, but we prefer to state here that politics should of course act in different areas as a complement to education, in order to meet the challenges of the Anthropocene.

Indeed, it is the technoscientific enterprise (Jonas 1990), with its metaphysically materialistic tendency taken over economically by capitalism (Hagège 2019), that is largely responsible for the problems associated with the Anthropocene. This is why effective solutions for sustainable life together on Earth in this context might perhaps emanate from alternative visions, from a diversity of traditions, particularly those that place spirituality at the forefront, because they can give a motivating meaning to operational changes that promote degrowth and help to accept them, as has been abundantly argued in the literature (ex: Myers 2009; Gumo et al. 2012; Gupta and Agrawal 2017; Jain and Jain 2019). We conceptualized education for responsibility as a secular way to favor spiritual development (Hagège 2020) by becoming more conscious and thus more responsible of ones’ own functioning in order to regulate it (Hagège 2014, 2019). In the field of health, spirituality is also defined as an awareness of human–environmental entirety (which is called “non-duality” in the MBER program; Hagège 2022): a connection to others and to nature, which favors a harmonious life. Meditation is, at the origin, a set of spiritual practices, which can increase several types of skill linked to ethics (see Section 1.2.2), and which has been customized to adapt to Western mentalities. Thus, placing the spiritual aim alongside the ethical one, thanks to secular meditation-based educational programs such as the MBER program, could be a further step towards developing transformational creativity, responsibility and, beyond that, wisdom in the service of concrete ethical solutions to the problems inherent in the Anthropocene.

## Figures and Tables

**Table 1:** Means at the pretest in both experimental (n = 48) and control (n = 36) groups.

Dependent Variable	Control		MBER	
	Mean	s.d.	Mean	s.d.
*Creativity*	*3.72*	*1.02*	*3.41*	*0.87*
Conv. C-S Cr.	3.55	1.48	2.89	1.28
Div. C-S Cr.	3.82	1.04	3.50	1.21
Conv. E-L Cr.	3.55	1.37	3.59	1.26
Diverg. E-L Cr.	3.97	1.19	3.72	1.15
*Responsibility*	*4.37*	*0.75*	*4.53*	*0.64*
SOOR	4.30	0.97	4.54	0.84
AOOR	4.30	1.22	4.33	1.10

*Note*. s.d.: standard deviation.

**Table 2:** Centered means at the post-test in both experimental and control groups and results of the one-way between-groups analyses of covariance, using pretest scores as covariates.

Dependant Variable	Control		MBER					
	Mean	s.d.	Mean	s.d.	F	df	*p*	η^2^_p_
*Creativity*	*−0.56*	*(0.86)*	*0.48*	*(0.96)*	*28.07*	*76*	*<.001*	*0.27*
Conv. C-S Cr.	−0.35	(1.16)	0.37	(1.47)	9.69	74	.003	0.12
Div. C-S Cr.	−0.75	(1.12)	0.60	(1.20)	25.03	76	<.001	0.25
Conv. E-L Cr.	−0.63	(1.40)	0.56	(1.22)	14.72	70	<.001	0.17
Diverg. E-L Cr.	−0.53	(1.15)	0.46	(1.33)	14.00	76	<.001	0.16
*Responsibility*	*−0.31*	*(0.67)*	*0.22*	*(0.67)*	*13.71*	*81*	*<.001*	*0.14*
SOOR	−0.41	(0.82)	0.31	(0.83)	15.22	81	<.001	0.16
AOOR	−0.07	(0.95)	0.02	(1.00)	0.20	81	.65	0.00

*Note*. Mean: centered mean; s.d.: standard deviation; df: degree of freedom. Based on Cohen (1988, cited by Pallant 2003, p. 181); η^2^_p_ = 0.01 corresponds to a small size effect, 0.06 to a moderate one, and 0.14 to a large one.

**Table 3:** Bivariate Pearson correlations between scores and subscores of interests at the pretest (95 ≤ n ≤ 105).

Dependent Variables	Diverg. C-S Cr.	Converg. E-L Cr.	Diverg. E-L Cr.	*Responsibility*	SOOR	AOOR
*Creativity*	*n.r.*	*n.r.*	*n.r.*	*0.05*	*0.09*	−*0.03*
Conv. C-S Cr.	0.44 ***	0.33 **	0.53 ***	*0.07*	0.10	−0.02
Div. C-S Cr.	1	0.23 *	0.51 ***	*0.08*	0.06	0.07
Conv. E-L Cr.		1	0.36 ***	−*0.04*	−0.03	0.00
Div. E-L Cr.			1	*0.04*	0.12	−0.13
Sense of R.					1	0.06

*Note*. *: *p* < .05; **: *p* < .01; ***: *p* < .001. *n.r*.: non relevant.

**Table 4:** Student *t* tests among both groups below and above the median Creativity score in the experimental group (n = 48).

Group	Dependent Variable	Pretest		Post-Test					
		Mean	s.d.	Mean	s.d.	t	df	*p*	d
Lower Half Crea-Pre	*Creativity*	*2.70*	*(0.56)*	*4.36*	*(0.71)*	*10.20*	*22*	*<.001*	*2.13*
	Conv. C-S Cr.	2.09	(1.02)	4.10	(1.31)	6.63	21	<.001	1.41
	Div. C-S Cr.	2.70	(0.88)	4.48	(0.99)	6.35	22	<.001	1.32
	Conv. E-L Cr.	3.28	(1.18)	4.56	(1.15)	3.75	17	<.001	0.88
	Diverg. E-L Cr.	3.04	(1.15)	4.40	(1.37)	4.51	22	<.001	0.94
	*Responsibility*	*4.48*	*(0.63)*	*4.49*	*(0.73)*	*0.05*	*22*	*.96*	*0.01*
	SOOR	4.32	(0.84)	4.45	(0.87)	0.68	22	.51	0.14
	AOOR	4.58	(0.77)	4.38	(10.12)	−0.90	22	.38	0.19
Higher Half Crea-Pre	*Creativity*	*4.13*	*(0.39)*	*4.94*	*(1.09)*	*3.42*	*22*	*<.001*	*0.71*
	Conv. C-S Cr.	3.64	(1.05)	4.68	(1.59)	2.93	21	.01	0.62
	Div. C-S Cr.	4.30	(0.92)	5.13	(1.32)	2.01	22	.06	0.42
	Conv. E-L Cr.	4.18	(0.96)	5.00	(1.27)	2.51	21	.02	0.53
	Diverg. E-L Cr.	4.39	(0.66)	5.04	(1.22)	2.23	22	.04	0.47
	*Responsibility*	*4.55*	*(0.67)*	*4.90*	*(0.55)*	*2.77*	*22*	*.01*	*0.58*
	SOOR	4.67	(0.81)	4.95	(0.73)	1.45	22	.16	0.30
	AOOR	4.17	(1.35)	4.65	(0.91)	2.08	22	.05	0.43

*Note*. s.d.: standard deviation. Given Cohen (1988, cited by Pallant 2003); d = 0.2 corresponds to a small size effect, 0.5 to a moderate one, and 0.8 to a strong one. Low Half and High Half Crea-Pre: median split of the Creativity score at the pretest in the experimental group.

**Table 5:** Conditional effect of the group (control vs. experimental) on Awareness and Sense of One’s Own Responsibility (at the post-test) for different values of the moderator (Convergent conceptual–semantic Creativity at the pretest).

Conv. S-C Cr.	Effect	s.e.	t	*p*	LLCI	ULCI
1.77	0.19	0.11	1.71	.091	−0.03	0.41
3.17	0.33	0.08	4.37	<.001	0.18	0.49
4.57	0.48	0.11	4.54	<.001	0.27	0.69

*Note*. s.e.: standard error. LLCI: lower limit confidence interval; ULCI: upper limit confidence interval. r^2^ increases due to the interaction between C-SC and the group: 0.04; F(1, 74) = 3.67; *p* < .06. Model summary: r^2^ = 0.26; F(3, 74) = 8.54, *p* < .001.

## Data Availability

The data presented in this study are available on request from the corresponding author. The data are not publicly available due to privacy.

## Appendix A. Items of the “Awareness and Sense of One’s Own Responsibility Scale” (ASOORS)

ASOOR is linked to the consciousness of the interdependence of phenomena: the one that goes from the subject to the world and that is therefore necessary for responsibility (the other component of interdependence, absent from the scale, corresponds to the way our environment conditions us).

From an initial pool of 16 items, we retained 10 items after factor analysis. Both subscales are issued from this factor analysis.

The scale points, from 1 to 6, are strongly disagree, disagree, somewhat disagree, somewhat agree, agree, and strongly agree, respectively.

**Subscale 1: *Sense of One’s Own Responsibility (SOOR).***

SOOR1. Whatever I do doesn’t really impact the world (R)

SOOR2. In general, I don’t care too much about the impact of my thinking on my environment. (R)

SOOR3. Most of the time when making a decision I think mainly about my personal interests. (R)

SOOR4. In general, I do not care too much about the impact of my actions on the environment (R)

SOOR5. I am concerned that my actions contribute to a better world.

SOOR6. My actions have an impact on my environment.

SOOR7. My thinking has an impact on my human or nonhuman environment.

**Subscale 2: *Awareness of One’s Own Responsibility (AOOR).***

AOOR1. I am aware of my limitations and “flaws”.

AOOR2. I am aware of the consequences of my limits and “defects” in my life.

AOOR3. I am aware of the consequences of my limits and “faults” in my environment and others.
